# A porcine model of osteosarcoma

**DOI:** 10.1038/oncsis.2016.19

**Published:** 2016-03-14

**Authors:** A Saalfrank, K-P Janssen, M Ravon, K Flisikowski, S Eser, K Steiger, T Flisikowska, P Müller-Fliedner, É Schulze, C Brönner, A Gnann, E Kappe, B Böhm, B Schade, U Certa, D Saur, I Esposito, A Kind, A Schnieke

**Affiliations:** 1 Chair of Livestock Biotechnology, Technische Universität München, Freising, Germany; 2 Department of Surgery, Klinikum Rechts der Isar, Technische Universität München, Munich, Germany; 3 Roche Pharma Research and Early Development, Pharmaceutical Sciences, Roche Innovation Center Basel, F. Hoffmann-La Roche Ltd., Basel, Switzerland; 4 Department of Medicine II, Klinikum Rechts der Isar, Technische Universität München, Munich, Germany; 5 Department of Pathology, Klinikum Rechts der Isar, Technische Universität München, Munich, Germany; 6 Department of Pathology, Bavarian Animal Health Service, Poing, Germany; 7 Institute of Pathology, Heinrich-Heine-University of Düsseldorf, Düsseldorf, Germany

## Abstract

We previously produced pigs with a latent oncogenic *TP53* mutation. Humans with *TP53* germline mutations are predisposed to a wide spectrum of early-onset cancers, predominantly breast, brain, adrenal gland cancer, soft tissue sarcomas and osteosarcomas. Loss of p53 function has been observed in >50% of human cancers. Here we demonstrate that porcine mesenchymal stem cells (MSCs) convert to a transformed phenotype after activation of latent oncogenic *TP53*^*R167H*^ and *KRAS*^*G12D*^, and overexpression of MYC promotes tumorigenesis. The process mimics key molecular aspects of human sarcomagenesis. Transformed porcine MSCs exhibit genomic instability, with complex karyotypes, and develop into sarcomas on transplantation into immune-deficient mice. In pigs, heterozygous knockout of *TP53* was sufficient for spontaneous osteosarcoma development in older animals, whereas homozygous *TP53* knockout resulted in multiple large osteosarcomas in 7–8-month-old animals. This is the first report that engineered mutation of an endogenous tumour-suppressor gene leads to invasive cancer in pigs. Unlike in *Trp53* mutant mice, osteosarcoma developed in the long bones and skull, closely recapitulating the human disease. These animals thus promise a model for juvenile osteosarcoma, a relatively uncommon but devastating disease.

## Introduction

Animal models of human cancers are crucial for the development of urgently needed diagnostic and therapeutic techniques. Genetically modified mice are widely used, but their small size and short lifespan preclude some preclinical studies. It is, for example, difficult to scale down radiological, thermal or surgical treatment of tumours, or perform longitudinal studies of tumour progression and remission, or longer-term response to therapy.

Mouse and human cancer biology also differ. Murine cells are more easily transformed *in vitro* than human cells,^1^ and the set of genetic events required for tumorigenesis is different.^2^ Mouse models may therefore not always provide the best representation of human disease.

Pigs are increasingly important in biomedicine and offer valuable complementary resources for cancer research. The number of pigs genetically modified to replicate human diseases has increased dramatically.^3^ Valuable models such as cystic fibrosis and diabetes are established.^4, 5^ Work is also proceeding towards genetically defined porcine cancer models, such as inactivation of *BRCA1* for breast cancer,^6^ mutation of *TP53*^7^ and, as we have reported, knockout and conditional activation of mutant *TP53*,^8^ latent oncogenic *KRAS* mutation^9^ and truncating mutations of *APC* to model colorectal cancer.^10^ The relevance of pig cancer models depends on how closely they resemble human disease. Porcine cancer biology is a new field and many fundamental questions remain open. Not least, does replication of single or combined human oncogenic mutation(s) in a pig have an equivalent effect on cell transformation and tumorigenesis?

Sarcomas are a group of tumours originating from the mesenchyme. They fall into two categories: those with disease-specific chromosomal translocations, and those with complex unstable karyotypes. Sarcomas of the second type, for example, fibrosarcoma, liposarcoma, chondrosarcoma and osteosarcoma, often have *TP53* and *RB1* mutations.^11, 12^ Consistent with this, transformation of human mesenchymal stem cells (MSCs) requires disruption of the p53 and Rb tumour-suppressor pathways, stabilisation of MYC, activation of oncogenic RAS and telomere maintenance.^13^ We report that targeted mutation of endogenous porcine *TP53* and *KRAS* in primary MSCs results in neoplastic transformation and tumorigenesis. Molecular analysis shows the process closely resembles human sarcomagenesis.

Osteosarcoma is a relatively rare solid tumour but the most common primary bone cancer. It predominantly affects young people and is highly malignant, requiring aggressive surgical resection and cytotoxic chemotherapy. Five-year survival for patients with metastatic osteosarcoma is only around 30%.^14^ We report that pigs with heterozygous and homozygous inactivation of *TP53* consistently develop osteosarcomas, providing a new model of osteosarcoma at human scale to understand and treat this devastating disease.

## Results

### Oncogenic modification of porcine MSCs

Human sarcomas frequently originate from MSCs, often initiated by lesions affecting the p53 tumour-suppressor pathway. Other alterations, including oncogenic *KRAS* mutations or *MYC* amplification, may occur later during tumorigenesis. To show that similar molecular events lead to sarcomagenesis in a porcine model, we mutated the endogenous *TP53* and *KRAS* genes in porcine MSCs.

**Figure 1:**
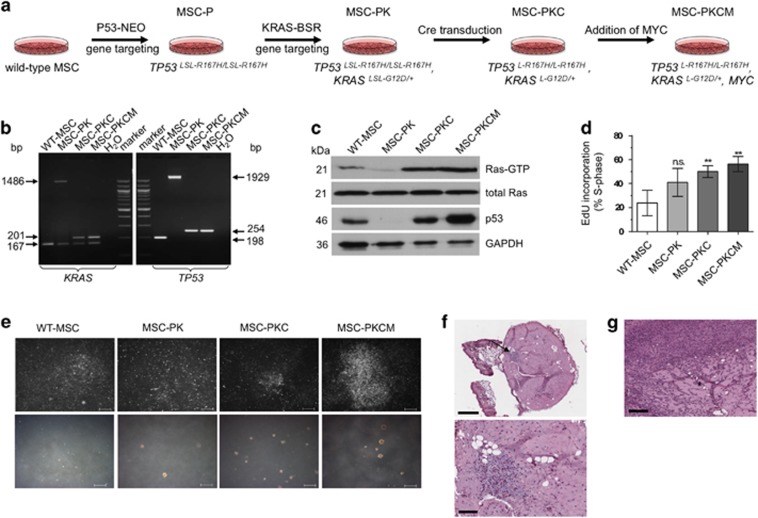
Transformation of porcine MSCs. (**a**) Overview of stepwise transformation of genetically modified porcine MSCs. (**b**) Left: Cre-mediated excision of transcriptional termination cassette from the *KRAS*^*LSL-G12D*^ allele. Cell types as shown. PCR amplification products across the site of the LSL-BS cassette in *KRAS* intron 1. Predicted fragment sizes: wild-type *KRAS* 167 bp; non-recombined *KRAS*^*LSL-G12D*^ 1486 bp; Cre-excised *KRAS*^*L-G12D*^ 201 bp. Right: Cre-mediated excision of transcriptional termination cassette from both *TP53*^*LSL-R167H*^ alleles. Cell types as shown. PCR amplification products across the site of LSL-NEO cassette in *TP53* intron 1. Predicted fragment sizes: wild-type *TP53* 198 bp; non-recombined *TP53*^*LSL-R167H*^ 1929 bp; Cre-excised *TP53*^*L-R167H*^ 254 bp. (**c**) Ras activation assay and p53 western blotting analysis. Cre-recombined MSC-PKC and MSC-PKCM cells show increased levels of active GTP-bound Ras proteins (21 kDa) as well as abundant levels of mutant p53-R167H proteins (46 kDa). (**d**) EdU (5-ethynyl-2'-deoxyuridine) incorporation during S phase. Data are consistent with the enhanced proliferative capacity of genetically modified derivatives relative to wild-type MSCs. *P*-values: MSC-PK=0.0727; MSC-PKC=0.0042; MSC-PKCM=0.0019. (**e**) Upper row: Loss of contact inhibition. MSC-PKCM cells form multi-layered foci when cultured at higher densities. Scale bars indicate 400 μm. Lower row: Anchorage-independent growth in soft agar. Wild-type MSCs grow as single cells in semi-solid medium, whereas MSC-PK, MSC-PKC and MSC-PKCM cells form three-dimensional colonies. Scale bars indicate 400 μm. (**f**) Upper: H&E-stained section of paucicellular nodule formed by injected MSC-PKC cells. The nodule consists mainly of matrigel with fat islands, capillary sprouts and isolated cells with slightly irregular nuclei. Scale bar indicates 500 μm. The arrow indicates an area of higher cellularity. Lower: Mesenchymal cells with slightly irregular nuclei embedded in a myxoid stroma are evident at higher magnification. Scale bar indicates 100 μm. (**g**) H&E-stained sections of MSC-PKCM derived tumour. This tumour is mostly highly cellular, but a matrix-rich area is still present, for example, as indicated by an asterisk. In the cellular areas, large tumour cells with pleomorphic nuclei are evident. A chronic inflammatory reaction is also present. Scale bar indicates 200 μm.

The porcine *TP53*^*R167H*^ mutation is orthologous to human *TP53*^*R175H*^. In a first round of gene targeting, we introduced a Cre-inducible latent mutant allele, *TP53*^*LSL-R167H*^, into porcine MSCs and derived one MSC cell clone homozygous for the mutant allele (*TP53*^*LSL-R167H/LSL-R167H*^), which in unrecombined form is a *TP53* knockout.^8^ For brevity, this genotype is referred to as MSC-P. It was subjected to a second round of gene targeting to introduce a G to A substitution into *KRAS* codon 12 (G12D) and a floxed transcriptional terminator cassette (LSL) into the first intron, generating the Cre-inducible allele *KRAS*^*LSL-G12D*^ (Supplementary Figures S1a–d). The resulting genotype (*TP53*^*LSL-R167H/LSL-R167H*^, *KRAS*^*LSL-G12D/+*^) is termed MSC-PK. One MSC-PK cell clone was transduced with Cre and subclones were isolated that had excised LSL cassettes from both mutant *TP53* alleles and the mutated *KRAS* allele (Figure 1b), termed MSC-PKC.

The *MYC* gene is often amplified in human cancers^15^ and has a pivotal role in transformation of human primary cells.^2, 16, 17^ We added a porcine MYC expression vector to one MSC-PKC cell clone and derived a pool (MSC-PKCM) that showed an average 1.7-fold increase in MYC mRNA expression and a similar increase in MYC protein (Supplementary Figures S2a and b). These genetic modifications are summarised in Figure 1a.

Reverse transcriptase PCR (RT–PCR) and sequence analysis showed that MSC-PKC and MSC-PKCM cells expressed mutant p53-R167H, wild-type KRAS and mutant KRAS-G12D mRNAs and biochemical evidence demonstrated increased levels of activated GTP-bound Ras and accumulation of mutant p53-R167H protein (Figure 1c), similar to their human and murine counterparts.^18, 19^

The progressive genetic modifications also resulted in phenotypic changes, such as accelerated cellular proliferation (Figure 1d), loss of contact inhibition in MSC-PKCM cells (Figure 1e top) and anchorage-independent growth (Figure 1e bottom).

### Genetically modified porcine MSCs form sarcomas in a xenotransplantation model

To analyse tumorigenic potential *in vivo*, porcine cells expressing mutant *TP53*^*R167H*^ and *KRAS*^*G12D*^ with and without additional *MYC* (MSC-PKC and MSC-PKCM) were xenografted into two immunodeficient mice. After 45 days, MSC-PKC cells formed a small nodule in one of the four injection sites with few atypical cells (Figure 1f). MSC-PKCM cells gave rise to tumours in three of the four injection sites in the other animal, classified as low-grade fibroblastic sarcomas (Figure 1g). Two such tumours were explanted and cultured to derive a porcine sarcoma cell line (poSARCO).

### Molecular analysis of transformed MSCs and sarcomas

**Figure 2:**
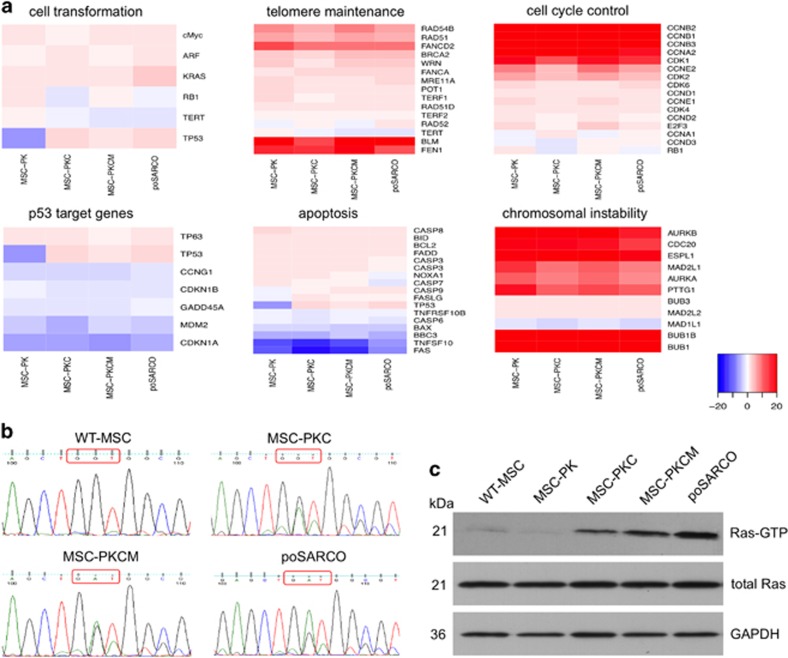
Expression profiles of cancer-related genes in transformed MSCs and sarcoma-derived tumour cells. (**a**) Differential gene expression heat maps relative to wild-type MSCs. Genes are grouped by functional categories, as indicated. The key at bottom right indicates fold-change values (log2 scale) represented as a colour gradient from blue (downregulation) to red (upregulation). See also Supplementary Table S2. (**b**) Sequence analysis of KRAS RT–PCR products amplified from exon 1 to exon 4. Cell types as shown. Codon 12 is indicated by a red box. RT–PCR products carrying the GAT sequence (G12D mutation) are the predominant species in poSARCO cells. (**c**) PoSARCO cells express higher levels of active GTP-bound Ras proteins (21 kDa) than the parental MSC-PKCM cells.

Microarray analysis was carried out to identify transcriptional changes associated with the stages of porcine MSC transformation. Figure 2a and Supplementary Table S1 show expression profiles for six groups of genes associated with cellular transformation: cellular transformation, telomere maintenance, cell cycle control, p53 target genes, apoptosis, and chromosomal instability (CIN).

#### Cellular transformation

*TP53* was not expressed in uninduced MSC-PK cells but upregulated after Cre activation. MYC expression increased in MSC-PKCM owing to exogenous expression. *KRAS* expression increased at each stage of cellular transformation and was the highest in poSARCO cells, likely owing to spontaneous gene amplification, as in many tumours.^20^ Quantitative PCR (Q-PCR) analysis revealed two *KRAS* copies in wild-type MSCs, MSC-PKC and MSC-PKCM, while poSARCO had six. Sequence analysis of KRAS mRNAs (exon 1 to exon 4) detected higher expression of KRAS-G12D than wild-type KRAS in poSARCO (Figure 2b). PoSARCO cells also exhibited increased levels of activated GTP-bound Ras protein (Figure 2c).

**Figure 3:**
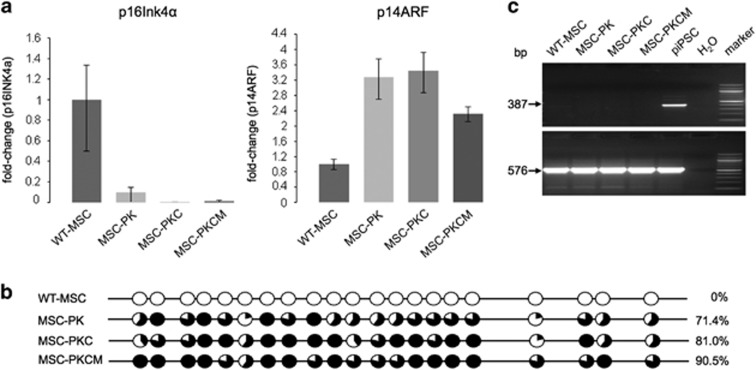
p16INK4, p14ARF and TERT expression. (**a**) Expression of porcine p16INK4α and p14ARF relative to wild-type MSCs. Cell types as shown. Values are normalised to GAPDH mRNA expression. Left: Downregulation of p16INK4α mRNA expression in stepwise modified MSC derivatives. Right: Increased p14ARF mRNA expression in modified porcine MSC derivatives. (**b**) CpG methylation analysis of the porcine *P16INK4A* locus (−10 to +256 bp relative to transcription start). Circles indicate methylation sites: filled circles represent methylated and open circles non-methylated sites. The proportion of total sites methylated is indicated at right. Cell types as shown. (**c**) Telomerase reverse transcriptase (TERT) expression is not activated in transformed porcine MSC derivatives. Porcine induced pluripotent stem cells (piPSC) are shown as a positive control for porcine TERT expression; a diagnostic 387-bp DNA fragment is evident. GAPDH was amplified (567 bp) as a control as indicated.

Primary human cells can escape cellular senescence through simultaneous inactivation of p53 and Rb.^21^ The *CDKN2A* gene products p16INK4α and p14ARF (p19ARF in mice) regulate the Rb and p53 pathways,^22, 23, 24^ but their roles differ between humans and mice^25^ and little is known about the pig. As shown in Figure 2a, *RB1* expression remained low in the MSC derivatives, but p14ARF (ARF) expression varied with the stages of cellular transformation. The microarrays used did not include p16INK4α, so p16INK4α and p14ARF expression was determined by Q-RT–PCR. Figure 3a shows that p16INK4α expression was diminished in MSC-PK cells, almost absent in MSC-PKC cells and similarly low in MSC-PKCM cells. Investigation of 21 CpG dinucleotides in the 5′ region of *p16INK4A* revealed that none were methylated in wild-type MSCs and progressively greater methylation in uninduced MSC-PK, activated MSC-PKC cells and the MSC-PKCM pool (Figure 3b).

#### Telomere maintenance

No reactivated *TERT* expression was observed (Figures 2a and 3c) in transformed MSC derivatives, but there was evidence for activation of the ALT mechanism, as frequently observed in human MSC-derived sarcomas.^26^ *BLM*, *FEN1*, *FANCD2* and, to a lesser extent, *FANCA* were upregulated. Genes involved in telomere elongation and capping, *RAD51*, *RAD54B* and *BRCA2*, were also upregulated, while shelterins *TERF1* and *TERF2* and *POT1* remained low, possibly indicating telomere deprotection (Figure 2a, Supplementary Table S1).

#### Cell cycle control

Loss of Rb function results in deregulated G_1_ checkpoint control and enhanced cellular proliferation. Although RB1 expression remained at low baseline levels, several cyclin genes and their interacting cyclin-dependent kinases were upregulated in the genetically modified MSCs and poSARCO cells (Figure 2a, Supplementary Table S1).

#### p53 target genes and genes associated with apoptosis

Reduced expression of the key feedback regulator *MDM2* indicates that mutant p53-R167H has impaired ability to transactivate its targets. This extends to genes important for cell cycle arrest, such as *CDKN1A*, *CDKN1B* and *GADD45A*, expression of which was lower than in wild-type MSCs and unaffected by induction of p53-R167H expression. Impaired capacity for apoptosis is indicated by lower expression of components of the death receptor pathway, for example, *FAS*, *TNFSF10* (TRAIL) and *TNFRSF10B* (KILLER/DR5); and of the mitochondrial pathway, for example, *BBC3* (PUMA), *BAX* and *CASP6* (Figure 2a, Supplementary Table S1).

#### Chromosomal instability

Several CIN-associated genes were strongly upregulated, including the spindle assembly checkpoint machinery genes *AURKA*, *AURKB*, *BUB1*, *BUB1B* and *MAD2L1*;^27, 28^ regulators of sister chromatid segregation *PTTG1* and *ESPL1*;^29^ and CDC20, a subunit of the anaphase-promoting complex/cyclosome, which is overexpressed in several human cancers and associated with poor prognosis^30^ (Figure 2a, Supplementary Table S1).

**Figure 4:**
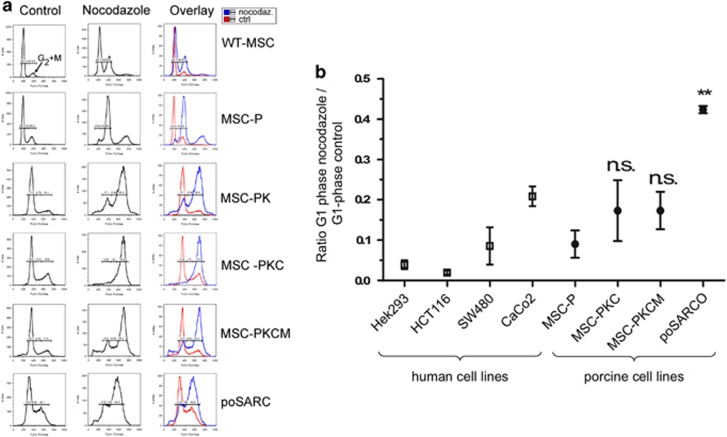
Chromosomal instability. (**a**) The number of cells in G_0_/G_1_, S and G_2_/M phase determined by flow cytometry, after growth under normal conditions (control), and 24 h after treatment with nocodazole. Representative DNA content histograms are shown for cell types as indicated. Wild-type MSCs show a strong increase in the G_2_/M-phase peak in response to nocadazole. In contrast, p53-deficient cell types (MSC-P, MSC-PK) and cells that express mutant p53-R167H (MSC-PKC,MSC-PKCM poSARCO) show no such increase but feature polyploid DNA peaks after nocodazole treatment. (**b**) Mitotic block index. The ratio of G_1_ peak in nocodazole-treated cells compared with control conditions (mean±s.d. for *n*=4 assays) shown for human cell lines and porcine MSC derivatives as indicated. A low index value indicates an effective mitotic checkpoint arrest after nocodazole treatment, as shown for human HEK293 (non-tumour origin) and HCT116 cells (colon cancer cells, chromosomally stable but DNA microsatellite unstable). In contrast, human SW480 and CaCo2 cells have high index values, indicating failure to induce G_2_/M arrest. MSC-P cells have intermediate index values, whereas MSC-PKC and MSC-PKCM clones, and especially poSARCO cells, have highly elevated index values, indicative of mitotic checkpoint deficiency. The tumour-derived poSARCO cells feature significantly increased index numbers, compared with p53-deficient MSC-P (*P*=0.0055), whereas MSC-PKC and MSC-PKCM cells do not differ significantly from MSC-P.

To investigate the dysfunction of mitotic checkpoints suggested by deregulated expression of cell cycle control genes, cells were treated with nocadazole to disrupt the mitotic spindle and induce mitotic arrest. The number of cells in G_0_/G_1_, S and G_2_/M phase determined by flow cytometry is shown in Figure 4a. Figure 4b shows the ratio between nocodazole treated and untreated cells in G_1_ phase. Human colon cancer cell lines known to have CIN and mitotic checkpoint deficiency (SW480, CaCo2) and chromosomally stable human cell lines of non-tumour origin (HEK293) or from colon cancer (HCT116, microsatellite unstable) provided comparisons. G_1_-peak ratios ranged from 0.05 in HEK293, which show the strongest blocking response to nocodazole, to 0.2 for CaCo2 cancer cells, which showed a weak response. Transformed porcine MSCs showed elevated ratios of cells in G_1_ phase after nocodazole treatment, similar to the CIN-positive cell lines SW480 and CaCo2, and poSARCO cells displayed an even higher ratio (Figure 4b).

In summary, these data suggest that porcine MSCs resemble human MSCs in requiring perturbation of the p53, Rb, KRAS and MYC signalling pathways combined with a spontaneous telomerase-independent immortalisation step to convert to a fully transformed phenotype.

### TP53 inactivation in pigs is sufficient for development of osteosarcomas

We determined whether pigs carrying the latent *TP53*^*LSL-R167H*^ mutation^8^ in heterozygous or homozygous form would spontaneously develop tumours. Animals were regularly observed for alterations in health and wellbeing, and individuals were slaughtered periodically for necropsy examination.

**Figure 5:**
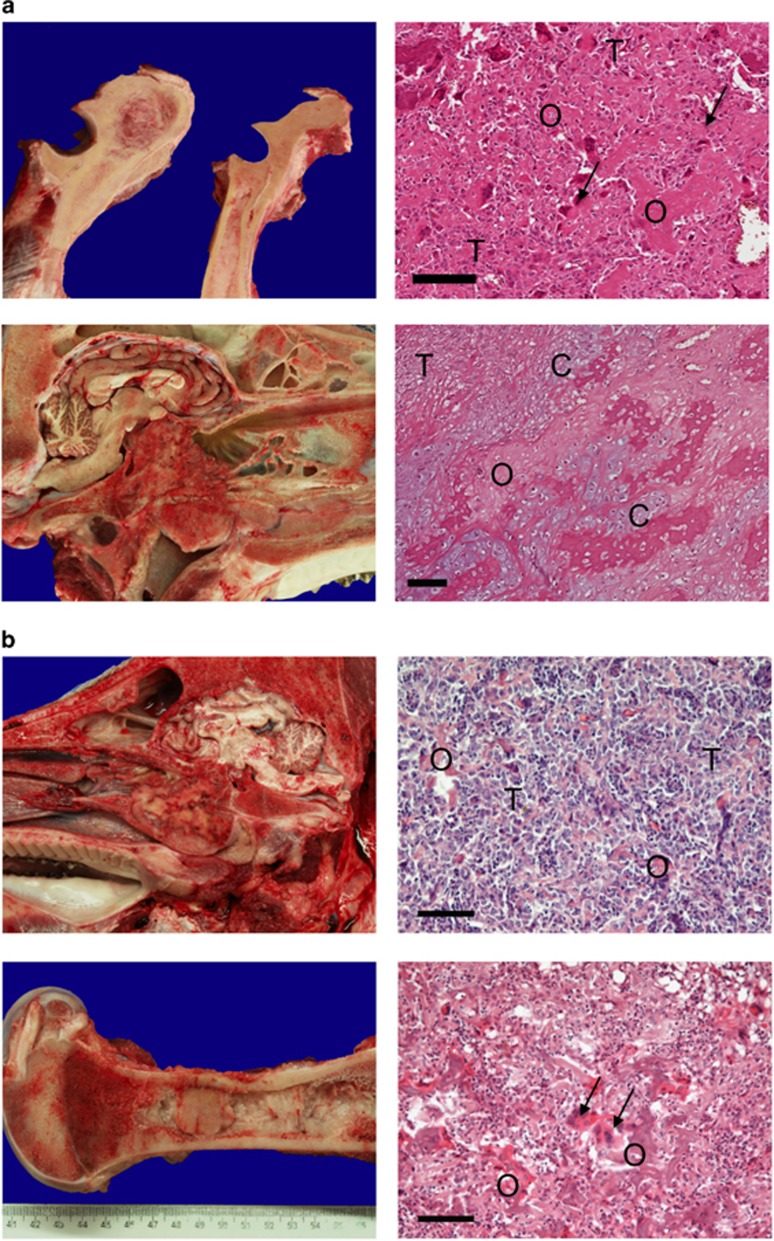
Osteosarcomas in *TP53* knockout pigs. (**a**) Upper: Osteoblastic osteosarcoma at left tuber olecrani in heterozygous knockout animal ID: 36. H&E-stained section shows tumour cells (T) with eccentric, hyperchromatic nuclei that produce osteoid (O). Multinucleate giant cells (arrows) with features of osteoclasts are scattered throughout the neoplasia. Scale bar indicates 100 μm. Lower: Osteoblastic and chondroblastic osteosarcoma at the skull basis in heterozygous knockout animal ID: 47. H&E-stained section shows tumour cells (T) that produce osteoid (O) and a chondroid matrix (C). Scale bar indicates 100 μm. (**b**) Upper: Osteoblastic osteosarcoma of the skull infiltrating os sphenoidale and vomer in homozygous knockout animal ID: 242. Polygonal tumour cells (T) and islands of osteoid (O). Scale bar indicates 100 μm. Lower: osteoblastic osteosarcoma in the bone marrow of left femur in homozygous knockout animal ID: 242. Multinucleated giant cells (arrows) were scattered throughout the neoplasia. Scale bar indicates 100 μm.

To date, nine heterozygous knockout pigs aged up to 32 months have been examined by necropsy, as summarised in Table 1. There was no evidence of tumours or other abnormalities in animals <16 months. Of the five older animals, four had tumours (ID: 34, 36, 47, 60), and one animal (ID: 49) had disseminated calcifying, ossifying lesions but no identifiable primary tumour. Figure 5a shows gross morphology and histology of tumours from two heterozygous and one homozygous animal.

Lesions from three pigs (animal ID: 34, 36, 47) were determined by histology as osteoblastic osteosarcomas. Two animals (animal ID: 34, 36) had osteosarcomas within long bones (metaphyseal region of the tibia and tuber olecrani) (Figure 5a top). The other animal (animal ID: 47) had a tumour in the nasal conchae, classified as an osteoblastic and chondroblastic osteosarcoma (Figure 5a bottom). Animal 60 showed a calcified fibrous tumour of the mandible.

We examined two F2 generation homozygous *TP53*^*LSL-R167H/LSL-R167H*^ knockout pigs (animal ID: 242, 336). These both grew more slowly than wild-type and heterozygous siblings, weighing 85–90 kg at 8 months while normal weight for this age and breed is 120–130 kg. Other than reduced size, neither showed ill effects or signs of distress in early life, but at 8 months pig 242 quickly lost condition and exhibited laboured breathing, and pig 336 developed paralysis of the hind legs. Both were slaughtered. Necropsy of pig 242 revealed multifocal osteoblastic osteosarcoma in the skull, left femur, left tibia, left humerus and pelvic bone. Figure 5b shows gross morphology and histology of the skull and left femur. Necropsy of pig 336 revealed multifocal osteoblastic osteosarcoma in the vertebral column, right femur and right ulna. The eighth thoracic vertebra was fractured, probably causing the limb paralysis.

Over the past 6 years, similar necropsies have been performed on 60 wild-type pigs aged between 12 and 48 months at the Bavarian Animal Health Service, none of which displayed any tumours. Moreover, there have been very few reports of spontaneous osteosarcomas in wild-type pigs.^31, 32^

### Radiation resistance

**Figure 6:**
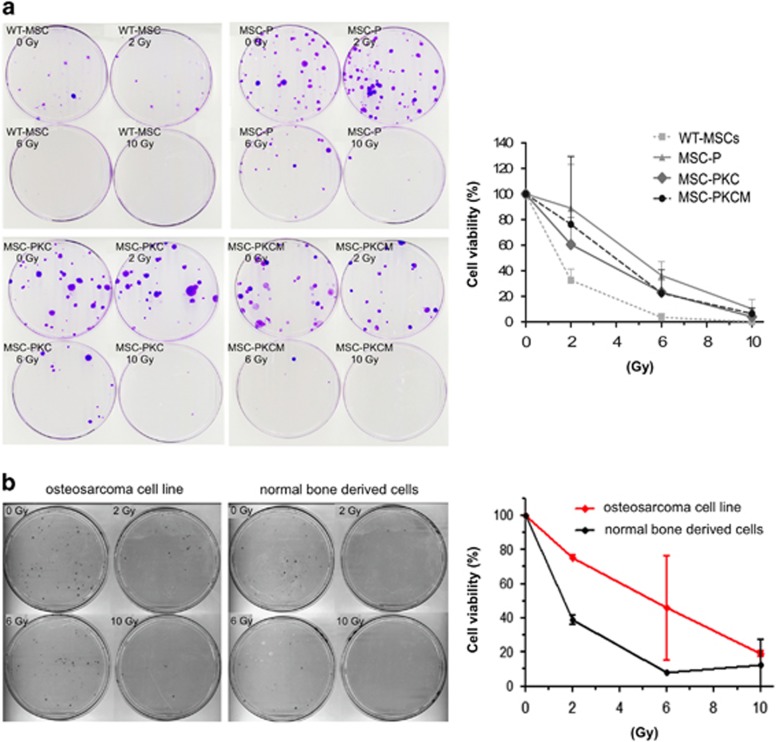
Transformed MSCs and osteosarcoma-derived cells show increased resistance to radiation. (**a**) Survival of cells after ^137^Cs irradiation after 2 weeks of culture. Left: Colonies stained with crystal violet; cell types and doses as indicated. Right: Mean survival±s.d., *n*=4. No significant differences were observed at 10 Gy irradiation. However, at 2 Gy transformed MSC showed increased viability compared with WT-MSCs: MSC-P (*P*=0.0184), MSCK-PKC (*P*=0.0500). MSC-PKCM cells did not attain significance (*P*=0.1558). Results were similar at the intermediate dose 6 Gy: MSC-P (*P*=0.0011); MSCK-PKC (*P*=0.0081); MSC-PKCM (*P*=0.0874). (**b**) Osteosarcoma cells show increased radioresistance. Cell survival in response to irradiation was assessed after 2 weeks of cell culture, as above. Cells were derived from a tumour on the left tibia of homozygous TP53 knockout pig (animal ID: 242) compared with cells from a tumour-free bone of the same animal. Tumour cells showed increased clonal growth after irradiation.

Several mutant p53 isoforms confer resistance to radiation.^33^ p53-deficient MSCs or MSCs expressing mutant p53 show increased resistance to ^137^Cs irradiation (Figure 6a). Similarly, cells derived from an osteosarcoma on the left tibia of homozygous *TP53* knockout pig 242 showed increased radioresistance compared with control cells derived from a tumour-free bone of the same animal (Figure 6b). Tumour-derived and normal cell isolates were characterised as positive for osteocalcin, osteonectin, type 1 collagen, alkaline phosphatase and vimentin and negative for EpCam.

### Multinucleation and CIN

*TP53* mutations are associated with CIN and human osteosarcomas have genome-wide DNA instability.^34^ Osteosarcoma cells isolated from *TP53* heterozygous pig 47 and *TP53* homozygous knockout pig 242 were checked for mitotic abnormalities, as were the p53-deficient MSC clones (MSC-P, MSC-PK), cell clones with activated *TP53-R167H* mutation (MSC-PKC, MSC-PKCM) and tumour-derived poSARCO cells. Multinucleated cells with fragmented nuclei were common in all samples (Supplementary Figures S3a and b). Many osteosarcoma-derived cells showed nuclear abnormalities, such as giant nuclei, multinucleated cells, micronuclei, lagging chromosomes (anaphase bridges) and abnormal spindle apparatus (Supplementary Figures S3b and c).

Chromosome counting also revealed that only 34.7% of metaphase spreads (*n*=174) from osteosarcoma cells (homozygous *TP53* knockout pig 242) were karyotypically normal, compared with 77% from wild-type MSCs (*n*=100).

## Discussion

*In vitro* studies of sarcomagenesis with human and mouse MSCs revealed that murine MSCs can be transformed by abrogation of p53 function alone.^35^ Human MSCs are more refractory, requiring disruption of both the Rb and p53 pathways to bypass senescence in combination with ectopic expression of TERT, oncogenic HRAS^V12^ and SV40 small T antigen.^13^ We report that porcine MSCs resemble human MSCs, requiring perturbation of p53, KRAS and MYC signalling pathways with spontaneous Rb pathway inactivation and telomerase-independent immortalisation steps to convert to a fully transformed phenotype. Immortalisation via the ALT mechanism rather than telomerase reactivation accords with findings from human mesenchymal malignancies.^26^

There have been two other reports of neoplastic transformation of porcine primary cells *in vitro*, based on overexpression of randomly integrated oncogenic transgenes,^36, 37^ but these are generally expressed at non-physiological levels. Our findings are based on targeted mutation of endogenous genes, more closely mimicking natural lesions that underlie cancer.

We demonstrate that null mutation of *TP53* results in spontaneous osteosarcomas in both heterozygous (>20 months) and homozygous (7–8 months) form. Rapid disease onset is an important practical advantage for future use of this model and compares well with mice, where homozygous *Trp53* knockout animals develop neoplasms around 6 months of age.^38^ The natural lifespan of a pig is approximately 10–15 years.

The effects of osteosarcoma and the necessary surgery can be devastating. There is an urgent need for animal models to improve methods of surgical management, develop new drugs and understand the molecular basis of disease initiation and progression. Most osteosarcomas are sporadic and of unknown cause but can be induced by radiation treatment. They frequently have *TP53* and *RB1* mutations^34, 39^ and alterations affecting cell cycle checkpoints, such as reduced p16INK4A expression.^40^ Patients with Li-Fraumeni syndrome and hereditary retinoblastoma are predisposed to develop osteosarcomas.^41, 42^ Disruption of p53 often leads to genomic instability, defective nocodazole-induced mitotic spindle checkpoints and resistance to radiation, all of which we observed. We showed that cells derived from porcine osteosarcomas are resistant to radiation, consistent with radioresistance of human osteosarcomas. Similar to human osteosarcoma cell lines and tumour samples from patients, porcine osteosarcoma cells displayed nuclear abnormalities and atypical mitotic figures (anaphase bridges, abnormal spindle apparatus). Moreover, metaphase spreads of porcine osteosarcoma-derived cells displayed highly variable chromosome numbers, again similar to human data.^43^ As up to 50% of human osteosarcomas have alterations in the *TP53* gene^44^ and various cytogenetic abnormalities,^45^ we consider that our porcine osteosarcoma model provides a valuable platform for studying such genetic changes.

Genetically modified mice have so far been the natural focus for modelling osteosarcoma. Germline *Trp53* inactivation in mice results in diverse cancers, with ~25% osteosarcomas in heterozygotes and ~4% osteosarcomas in homozygotes, which mainly develop lymphomas.^46^ The high incidence of other tumours is a problem that has motivated development of improved mouse osteosarcoma models with Cre-mediated conditional deletion of *Trp53* in the osteogenic lineage, sometimes in combination with *Rb1*. These models show highly penetrant osteosarcoma formation but have been criticised because murine primary tumours predominantly affect the axial skeleton, while human osteosarcomas are most common in the long bones of the limbs.^47^

Work in rats is less advanced than in mice, but a similar mixed tumour spectrum has been reported, with approximately half of heterozygous *Trp53* knockout rats developing osteosarcomas, while most homozygotes develop haemangiosarcoma.^48^

Yucatan pigs have been described that carry the *TP53*^*R167H*^ mutation.^7^ Pigs heterozygous for this mutant allele were reported to be free of tumours up to 30 months, while homozygous pigs mainly developed lymphomas and some osteogenic tumours. These findings differ from ours, perhaps owing to the different breed, the p53 mutation or both.

Many different p53 mutations have been analysed in humans and mice. Human *TP53*^*R175H*^ (orthologous to porcine *TP53*^*R167H*^) is thought to impart gain-of-function properties with dominant-negative effect. Mutant p53-R175H inhibits wild-type p53 interaction with promoter elements,^49^ advances angiogenesis,^50^ and promotes epithelial–mesenchymal transition.^51^ Genotype–phenotype analysis of Li-Fraumeni families revealed that patients carrying such a mutation in the core p53 DNA-binding domain show more highly penetrant cancer phenotypes with higher incidence and earlier onset than those with *TP53* inactivation mutations.^52, 53^ These observations are also reflected in mouse studies. Mice heterozygous for *Trp53*^*R172H*^ (orthologous to human *TP53*^*R175H*^ and porcine *TP53*^*R167H*^) developed more tumours and a different tumour spectrum than heterozygous knockout mice.^54^

We previously showed that p53-deficient porcine cells develop a transformed phenotype with more rapid cell doubling, growth in semi-solid medium and resistance to the chemotherapeutic drug doxorubicin and that these characteristics were more pronounced in porcine cells that express mutant p53-R167H,^8^ suggesting that porcine p53-R167H is a gain-of-function mutation, as per murine and human data. An important advantage of our model is that the *TP53* knockout allele can be activated to express mutant p53-R167H, so it will, in future, be possible to generate animals that express p53-R167H for direct comparison with p53 inactivation in the same breed.

To the best of our knowledge, ours is the first report of *TP53* knockout pigs. The anatomical locations of osteosarcomas observed are mainly the long bones, skull and mandible. Analysis of homozygous p53 deficiency is so far restricted to two animals but indicates accelerated tumour development and, unlike rodents, no change in tumour spectrum relative to heterozygous knockout.

Our priority is now to further investigate how accurately the model represents the human disease. The initiation and progression of human osteosarcoma are not well understood and we hope the porcine model will help elucidate the molecular pathways and driver mutations involved. Imaging studies will enable non-invasive longitudinal investigation of individual animals to identify early-stage tumours, analyse disease progression and investigate metastasis. Several practical advantages of the model are already apparent, not least its simplicity, with no need for tissue-specific gene inactivation or multiple engineered mutations. It is also helpful that osteosarcomas form relatively late in heterozygotes, allowing normal breeding and expansion of the herd, and with short latency in homozygotes that can be produced as required.

## Materials and methods

### Reagents

Chemicals were obtained from Sigma-Aldrich Chemie GmbH (Munich, Germany) unless otherwise specified; cell culture media and supplements were obtained from ThermoFisher Scientific (Waltham, MA, USA) or Invitrogen (Carlsbad, CA, USA) unless otherwise specified.

### Animals

Animal studies were approved by the Government of Upper Bavaria (permit number 55.2-1-54-2532-34-09) and performed according to the German Animal Welfare Act and European Union Normative for Care and Use of Experimental Animals and were approved by the Institutional Animal Care and Use Committees of Technische Universität München, and Regierung von Oberbayern.

Nine *TP53*^*LSL-R167H/+*^ and two *TP5*^*LSL-R167H/LSL-R167H*^ Landrace pigs aged 7.5–32 months, male and female, were generated and raised in our own facilities with food and water provided *ad libitum*.

Two 7- and 10-week-old female NOD.Cg-Prkdcscid Il2rgtm1Wjl/SzJ (NSG) mice were purchased from Jackson Lab (Bar Harbor, ME, USA) and maintained in specific pathogen-free facilities with food and water provided *ad libitum*.

### Necropsy examination and tumour analysis

Pigs were humanely killed and complete necropsy examination was carried out at the Tiergesundheitsdienst Bayern (Bavarian Animal Health Service). Specimens were fixed, embedded, sectioned and stained by standard methods. Bone specimens were first decalcified in Ossa Fixona (Waldeck GmbH, Münster, Germany).

### Porcine primary cells and cell lines

Porcine primary cells and cell lines were derived in house and regularly screened for mycoplasma contamination. Cell lines of human origin (Hek293, HCT1116, SW480,CaCo2) were obtained from ATCC or DSMZ (Braunschweig, Germany), cultured in Dulbecco's Modified Eagle's Medium (DMEM) with foetal calf serum (7%), 1% Pen/Strep and 1% glutamine and tested every 2 months for mycoplasma infection. To avoid contamination and phenotype changes, cells were kept as frozen stocks and cultured for 4 weeks maximum.

### Generation of TP53^R167H^, KRAS^G12D^ double gene targeted porcine MSC clones

Derivation and culture of porcine *TP53* gene-targeted MSC clones has been described previously.^8^ The vector KRAS-BSR (Supplementary Figure S1a) is the same as vector KRAS-NEO, which has been described previously,^9^ but contains the blasticidin resistance gene (*bsr*) instead of *neo*.

KRAS-BSR targeted cell clones were identified by: 5′ and 3′ junction PCR, RT–PCR, and restriction fragment-length polymorphism to confirm the G to A mutation, as described elsewhere.^9^ Cre-mediated induction of *TP53*^*R167H*^ and *KRAS*^*G12D*^ alleles was confirmed by PCR and RT–PCR analysis as described elsewhere.^8, 9^ All PCR primers used and diagnostic fragments amplified are shown in Supplementary Table S2.

### Porcine MYC expression vector

This vector comprised the mouse phosphoglycerate kinase promoter directing expression of 1.361 kb porcine *MYC* cDNA linked to 98 bp *MYC* 5′ and 178 bp *MYC* 3′ untranslated regions (NCBI accession number NM_001005154.1: 494…2128). A *bsr* selectable marker linked by an internal ribosome entry sequence was placed 3′ of the *MYC* coding sequence.

### Cre recombinase protein transduction

Cre protein was produced *in vitro* with the vector pTriEx-HTNC (Addgene plasmid 13763, Cambridge, MA, USA) as described^55, 56^ and transduced as described previously.^8^ After 96 h, cells were plated by limiting dilution into 96-well plates to derive single-cell clones.

### Bisulphite sequencing

In all, 500 ng genomic DNA samples were converted using the EpiTect Fast Bisulphite Conversion Kit (Qiagen, Germantown, MD, USA) by standard methods. A 266-bp *P16INK4A* fragment (NCBI accession number AJ316064.1: 299…565) was amplified using primers BS-p16F and BS-p16R with GoTaq Polymerase (Promega, Madison, WI, USA). Thermal cycling conditions were: 30 min, 95 °C; then 35 cycles of: 30 s, 95 °C; 30 s, 54 °C; 30 s, 72 °C; followed by 5 min, 72 °C. Amplified PCR products were processed as described previously.^57^

### Copy number variation analysis

Q-PCR was carried out using Fast SybrGreen PCR MasterMix and run on 7500 Fast Real-Time PCR System (both from Applied Biosystems, Foster City, CA, USA). Relative quantification of *KRAS* copy number was performed with primers CNV-KRASF and CNV-KRASR. *GAPDH* (glyceraldehyde 3-phosphate dehydrogenase) was amplified using primers CNV-GAPDHF and CNV-GAPDHR. Relative quantification of copy number alterations was performed in 10 μl with 10 ng genomic DNA. Thermal cycling conditions were 10 min, 95 °C; then 40 cycles of: 15 s, 95 °C; 1 min, 60 °C. Samples were run in triplicate, data were normalised to *GAPDH* and copy numbers were calculated by the ΔΔC_T_ method.

### Quantitative real-time RT–PCR

Relative quantification of gene expression was carried out by two-step Q-RT–PCR using Fast SybrGreen PCR MasterMix. Primers used were: p16INK4α RT-p16F and RT-p16R; p14ARF RT-p14F and RT-p14R; MYC RT-MycF and RT-MycR; and GAPDH RT-GAPDHF and RT-GAPDHR. Reactions were performed in 10 μl. Thermal cycling parameters were as above. Samples were assayed in triplicate, relative expression was normalised to GAPDH expression and fold-differences were calculated by the ΔΔC_T_ method.

### Western blotting analysis

Protein lysates were prepared as described previously.^8^ Total protein (40 μg) was loaded in each lane and separated by 15% sodium dodecyl sulphate–polyacrylamide gel electrophoresis. Blotting and detection of p53 and GAPDH was as described previously.^58^ Porcine MYC was detected with anti-c-MYC (*N*-262, Santa Cruz Biotechnology, Heidelberg, Germany) diluted 1:250.

### Ras activation assay

Ras activity was analysed using the Ras Activation Assay Kit (Merck, Millipore, Darmstadt, Germany). Total protein (1 mg) was incubated with 10 μg Raf-1 RBD agarose for 3 h at 4 °C with gentle agitation. Western blotting and detection were carried out according to the manufacturer's instructions.

### Growth characteristics of porcine cells

Cycling and non-cycling cells in the G_0_/G_1_ peak were differentiated by 2-h pulse-labelling with 5-ethynyl-2'-deoxyuridine and then staining with the Click-iT EdU Flow Cytometry Assay Kit (ThermoFisher). For the focus formation assay, 1 × 10^5^ cells were plated onto 10-cm cell culture dishes and cultured for 4 weeks, and the medium was changed every 2–3 days. Anchorage-independent growth assay of primary and transformed porcine cells was carried out as described.^8^

### Tumour growth and isolation of porcine sarcoma-derived tumour cells

Groups of 1 × 10^7^ cells suspended in chilled DMEM were mixed 1:1 with high concentration matrigel basement membrane matrix (BD Biosciences, Bedford, MA, USA) and implanted subcutaneously into NSG mice. Mice were humanely killed, tumours excised, fixed, sectioned and H&E (haemotoxylin and eosin) stained by standard methods. Cells were derived from a minced piece of tumour digested in DMEM supplemented with 200 U/ml collagenase type IV (Worthington, Lakewood, NJ, USA) at 37 °C for 24 h, then centrifuged at 100 r.c.f. for 5 min. Cells were resuspended in standard porcine MSC medium,^8^ cultured at 37 °C, 5% CO_2_ and passaged at regular intervals.

### Establishment of cell cultures from porcine osteosarcoma

Cell cultures were established from osteosarcomas as described above for tumour xenografts, cultured and passaged by standard methods.

### Gene expression profiling and data analysis

cDNA was prepared from total RNA by standard methods. Gene expression experiments were conducted on custom *Sus Scrofa* 12-plex microarrays containing 142 073 probes targeting 17 261 genes. Probe selection and array design is described elsewhere.^59^ In all, 200 ng double-stranded cDNA was Cy3-labelled using the 'One colour DNA Labelling Kit' (Roche NimbleGen, Madison, WI, USA). A minimum of three technical replicates of each sample were loaded, and raw data intensities were collected using a NimbleGen MS200 scanner. Background corrected, quantile normalised and RMA (Robust Multi-Array Analysis) normalised probe intensities were generated using the NimbleScan v2.6 software (Roche NimbleGen). Average signal intensities were calculated for each probe set and log2 transformed. Data were analysed using the Partek Genomics Suite software, v6.6 (Partek, St Louis, MO, USA). Significant differentially expressed genes were identified for a false-discovery rate *P*-value of 0.05 and twofold change.

### Indirect immunofluorescence staining

Immunostaining and fluorescence micrography were performed as described previously.^60^ Antibodies and reagents used were: anti-α-tubulin (MABT205, Calbiochem, San Diego, CA, USA), 4′,6-diamidino-2-phenyl indole (DAPI) and TRITC-Phalloidin (P1951, Sigma-Aldrich), secondary antibodies coupled to fluorophores (Jackson Immunoresearch, West Grove, PA, USA).

### Cell cycle analysis

Mitotic arrest was induced with 100 ng/ml nocadazole for 20 h. Cells were harvested, washed, resuspended in ice-cold Ca^2+^/Mg^2+^-free phosphate-buffered saline with 1 mg/ml glucose, fixed in 70% ice-cold ethanol overnight at 4 °C and incubated 1 h at room temperature in the dark with 50 μg/ml propidium iodide and 0.4 mg/ml RNaseA (Qiagen). Cells were washed, resuspended in phosphate-buffered saline with 0,5% (v/v) bovine serum albumin and 0.01% (w/v) NaN_3_ and strained before fluorescence-activated cell sorting analysis. Cell cycle profiles were measured using a FACSCalibur device (BD Biosciences, San Jose, CA, USA) with the CellQuest Pro software (BD Biosciences). Doublets, dead cells and debris were excluded, and single-parameter DNA histograms were analysed with the FlowJo software (Tree Star, Ashland, OR, USA) to identify G_1_ and G_2_/M peaks and the S-phase populations from univariate distribution curves.

### Cell irradiation clonogenic assay

Aliquots of 5 × 10^5^ cells were irradiated with 2, 6 and 10 Gy from a ^137^Cs source (GE Healthcare Buchler, Braunschweig, Germany), seeded in six-well plates and cultured under standard conditions overnight. A total of 1 × 10^2^ cells were seeded on 10-cm dishes and cultivated under standard conditions for 14 days. Plates were fixed and stained with 0.1% crystal violet by standard methods, and colonies were counted by two observers.

### Statistical analyses

Statistical analyses were performed using GraphPad Prism 5 and GraphPad InStat3 (GraphPad Software, La Jolla, CA, USA). Data are presented as mean±s.d. Comparison between two data sets was carried out using Student's unpaired *t*-test, after prior testing for Gaussian distribution. Non-normally distributed sample sets were tested with Mann–Whitney *U*-tests. All statistical comparisons were carried out at a 0.05 threshold level of significance.

### Karyotype analysis

Primary porcine cells were treated with 0.8 μg/ml colcemid for 20 h, dissociated with 10 × trypsin/EDTA, treated with 0.8% Na-citrate/75 mm KCl/H_2_O (1:1:1) and fixed with ice-cold methanol/acetic acid (3:1). Metaphase spreads were treated with 0.03 mg/ml pepsin in 0.01m HCl, 45 s at 37 °C, DAPI stained and mounted with antifade.

## Figures and Tables

**Table 1:** Necropsy examination of heterozygous and homozygous TP53 knockout pigs

*Age (months)*	*ID*	*TP53*	*Gen.*	*Tumour pathology*	*Tumour location*	*Tumour dimensions*
10	56	Heteroz. KO	G0	None detected		
12.5	64	Heteroz. KO	G0	None detected		
14	48	Heteroz. KO	G0	None detected		
16	45	Heteroz. KO	G0	None detected		
21	49	Heteroz. KO	G0	None detected Multifocal calcifying and ossifying fibroplasia on liver capsule, in mesentery (plica ileocaecalis) and in lymph nodes (Lnn. ileocolici).		
21.5	60	Heteroz. KO	G0	Calcifying fibrous tumour Nodular neoplasm consisting of a dense poorly demarcated cell population with infiltration into surrounding tissue. Closely packed cells formed bundles and streams in a fibrous stroma with multiple foci of collagen bundles with dystrophic calcification. Cells were spindled with indistinct cell borders and showed a moderate amount of eosinophilic, fibrillar cytoplasm and an oval-to-spindled nucleus with either sparsely or densely packed chromatin and one or two nucleoli. Anisocaryosis and scattered multinucleated giant cells present. Mitotic activity low. Additionally, vascular and capsular invasion, focal necroses and haemorrhages, as well as purulent and ulcerating inflammation.	Mandible. Corpus mandibulae, pars incisiva, bone marrow and compacta	6 × 5 × 4 cm^3^
21.5	34	Heteroz. KO	G0	Osteoblastic osteosarcoma Lobular neoplasm consisting of a dense cellular, well-demarcated cell population with infiltration into surrounding tissue. Two cell morphologies were distinguishable. In some parts, sheets of neoplastic cells in a loosely arranged fibrous stroma with foci of eosinophilic amorphous osteoid. The polygonal cells with indistinct cell borders had a moderate amount of eosinophilic cytoplasm and a polygonal eccentric nucleus with either sparsely or densely packed chromatin and one or two nucleoli. In other parts, bundles and streams of neoplastic cells in a fibrous stroma with osteoid foci. Spindled cells with indistinct cell borders had a moderate amount of eosinophilic cytoplasm and an oval-to-spindled nucleus with either sparsely or densely packed chromatin and one or two prominent nucleoli. In all parts, cells showed anisocaryosis and anisocytosis and scattered multinucleated giant cells. Mitotic activity varied from low to high. Vascular and capsular invasion, focal necroses and haemorrhages.	Left tibia. Proximal metaphysis, bone marrow, compacta, surrounding soft tissue	ø 4 cm
				Cavernous haemangioma Well-circumscribed proliferation in the dermis consisting of dilated blood-filled spaces in very little stroma. Vascular spaces were lined by well-differentiated endothelial cells. No mitoses visible.	Skin. Inner claw, left hind limb	ø 0.5 cm
23	47	Heteroz. KO	G0	Osteoblastic and chondroblastic osteosarcoma Similar morphology as animal ID 34. Spindle cell type predominated. Scattered foci of cartilage tissue present.	Skull. Os ethmoidale and vomer	6.5 × 3.5 × 6.5 cm^3^
32	36	Heteroz. KO	G0	Osteoblastic osteosarcoma Similar morphology as animal ID 34. Polygonal cell type predominated.	Left ulna. Tuber olecranon, bone marrow and compacta	ø 5 cm
8	242	Homoz. KO	F2	Multifocal osteoblastic osteosarcoma Similar morphology as animal ID 34. Spindle cell type present in pelvic neoplasia. Polygonal cell type dominated the neoplasia in skull and extremities. Focal purulent inflammation and focal lymphocyte infiltration.	Skull. Os sphenoidale and vomer	6 × 4 × 4 cm^3^
					Left femur. Diaphysis, bone marrow	2 × 2 × 2 cm^3^
					Left tibia. Proximal epiphysis, compacta	6 × 2 × 2 cm^3^
				Hyperplasia of bone tissue in the diaphysis of both ulnae and right femur Multifocal calcifying and ossifying fibroplasia in mesentery (plica ileocaecalis)	Left humerus. Diaphysis, bone marrow	6 × 2 × 2 cm^3^
					Pelvic bone, right os ilium, bone marrow	ø 1.5 cm
7.5	336	Homoz. KO	F2	Multifocal osteoblastic osteosarcoma	Eighth thoracic vertebra. Corpus vertebrae	5.5 cm, ø 0.5 cm
				Similar morphology as animal ID 34. The spindle cell type dominated. The tumour mass proliferated into the vertebral canal and compressed the spinal cord over a distance of 5.5 cm.	Right femur. Diaphysis	2.8 × 2 × 1.2 cm^3^
					Right ulna. Epiphysis and diaphysis	2 × 2 × 1 cm^3^

Abbreviations: Gen., generation; heteroz. KO, *TP53*^*LSL-R167H/+*^ pigs; homoz. KO, *TP53*^*LSL-R167H/LSL-R167H*^ pigs.
