# Supplementary material for: A porcine model of osteosarcoma
Source: Oncogenesis. 2016 Mar 14;5(3):e210–. doi: 10.1038/oncsis.2016.19 (PMC4815050; doi:10.1038/oncsis.2016.19)
Supplement: Supplementary Information [file oncsis201619x1.doc]

**Supplementary Figures**

**Supplementary Figure 1. Porcine *KRAS^LSL-G12D^*gene targeting using KRAS-BSR construct**

a, upper: Porcine *KRAS* gene locus. Exons are numbered, coding and non-coding exons are marked in black and open boxes.

lower: KRAS-BSR gene targeting vector. Vector presents regions of homology, the transcriptional termination cassette (LSL) in intron 1 and the G to A point mutation (encoding G12D) in exon 2 is indicated by an asterisk. PCR as well as RT-PCR primers that were used to identify and analyse gene targeted cell clones.

b, Upper panel shows PCR detection of a diagnostic 3.4 kb fragment over the *KRAS^LSL-G12D^* 5’ junction site. Lanes show wild-type MSCs, the p53 deficient cell clone MSC-P and KRAS-BSR transfected MSC-PK subclones (-39, -67, -82, -83 and -84).

The integration of the LSL-BS cassette at the targeted *KRAS* gene locus is indicated by the presence of a diagnostic 3.4 kb PCR product. Middle panel: Amplification of a diagnostic 3.3 kb fragment from the wild-type *KRAS* allele was carried out as loading control. The lower panel depicts the PCR amplification of a diagnostic 10.8 kb fragment across the 3’ junction of the targeted site. WT-MSCs and the parental cell clone MSC-P were included as negative controls.

c, *BccI* restriction fragment length polymorphism analysis to detect the G12D mutation in exon 2. *BccI* restriction digestion of a 2.8 kb PCR fragment covering exon 2 results in a diagnostic 391 bp DNA fragment containing wild-type codon 12. The presence of the G to A mutation at codon 12 leads to an additional *BccI* restriction enzyme recognition site resulting in 257 bp and 134 bp DNA fragments. Shown are KRAS-BSR transfected MSC-PK subclones (-39, -67, -82 and -84). RFLP for wild-type *KRAS*: 609, 520, 430, 391, 249, 190, 189, 187, 37, 31 bp; RFLP for mutant *KRAS^G12D^*: 609, 520, 430, 257, 249, 190, 189, 187, 134, 37, 31 bp

d, Expression of truncated KRAS-BS mRNA species. The upper panel shows RT-PCR detection of 536 bp truncated KRAS-BS transcripts (*KRAS* exon 1 to *bsr*) terminating in the LSL-BS cassette in: wild-type MSCs, p53 deficinet cell clone MSC-P and KRAS-BSR transfected cell clones MSC-PK39, MSC-PK67 and MSC-PK82. The lower panel illustrates RT-PCR detection of wild-type KRAS mRNA species (492 bp).

**
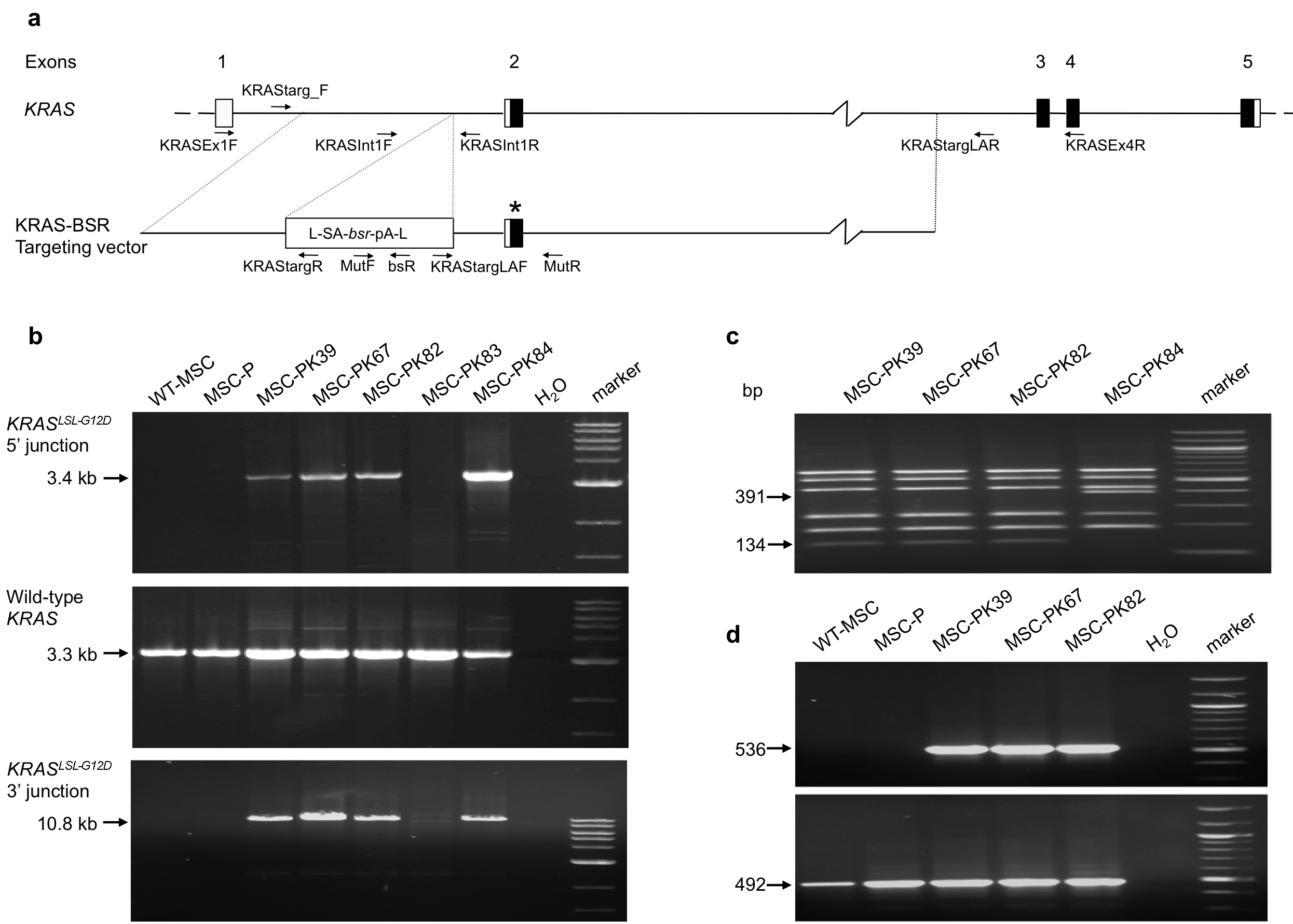
**

**Supplementary Figure 2. Increased expression of MYC in MSC-PKCM**

**a,** Relative MYC expression levels in stepwise modified porcine cells compared to their relative parental cell clone. Depicted are fold-change values of the cell clones, normalised to GAPDH expression. Stable selected MYC transfectants (MSC-PKCM) show an averaged 1.7 fold increase in MYC mRNA expression compared to untreated MSC-PKC cells.

**b,** Western blot analysis. Stable selected MYC transfectants (MSC-PKCM) show increased MYC protein levels.

**
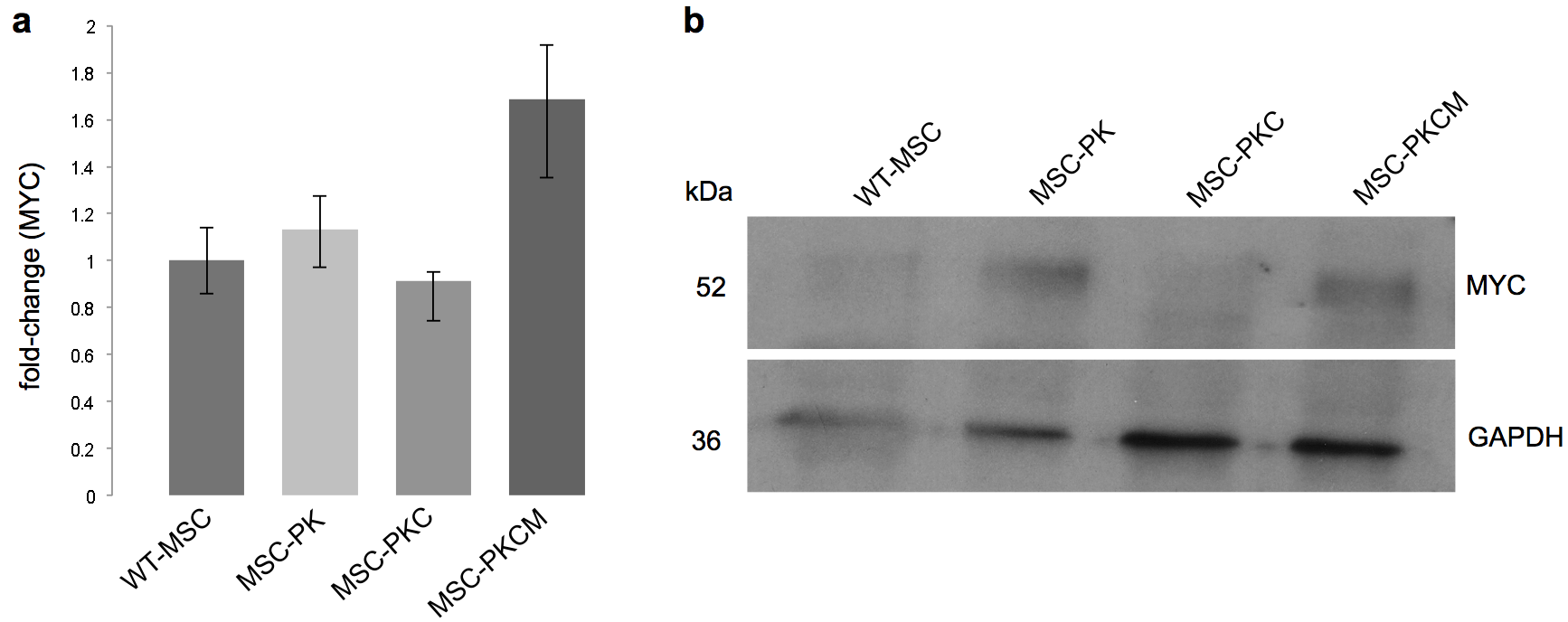
**

**Supplementary Figure 3. Multinucleation and chromosomal instability in stepwise modified mesenchymal stem cells and porcine sarcoma-derived tumour cells**

**a**, Cellular morphology was assessed by immunofluorescence microscopy analysis of microtubules (anti-tubulin staining, green), actin cytoskeleton (TRITC-phalloidin staining, red), nuclear staining (DAPI, blue), combined with phase contrast images (right row).

**Top row,** Wild-type MSCs show normal spindle-cell like cell morphology and no evidence of aberrations. MSC derivatives that lack p53 (MSC-P, MSC-PK) feature abnormal nuclei, increased nucleoli and multinucleated, enlarged cells. MSC derivatives that express mutant *TP53^R167H^* and *KRAS^G12D^* (MSC-PKC, MSC-PKCM) also show multinucleation, presence of micronuclei, and enlarged cell diameter (size bar: 20 µm). Porcine sarcoma-derived cells (poSARCO) feature enlarged atypical nuclei and increased nucleoli, as well as frequent micronuclei (bottom row, size bar: 10 µm).

**b,** Primary culture of osteosarcoma cells from *TP53* heterozygous knockout pig 47 and from *TP53* homozygous knockout pig 242 . Cellular morphology was assessed by immunofluorescence microscopy analysis of microtubules (anti-tubulin staining, green), actin cytoskeleton (TRITC-phalloidin staining, red), nuclear staining (DAPI, blue), combined with phase contrast images.

**Top row,** Morphology of interphase cells from *TP53* heterozygous knockout pig (animal ID:47) indicates prominent lamellipodia formation with strongly enriched F-actin at the leading edge. Nuclear atypia, with bi- and polynucleated cells and micronuclei (arrows), was frequently observed, even after short culture periods (48h). Aberrant mitotic figures with tripolar anaphase spindles are indicative of chromosomal instability. (Size bar: 20 µm).

**Bottom row**, Primary culture of osteosarcoma cells from *TP53* homozygous knockout pig (animal ID: 242) indicates aberrant nuclear morphologies and presence of micronuclei (arrows).

c, Nuclear atypia in primary osteosarcoma cells derived from *TP53* homozygous knockout pig 242. Of note, nuclear atypia was frequently observed (giant nuclei, multinucleated cells, micronuclei). Tumour cells showed signs of aberrant mitosis, presenting lagging chromosomes in anaphase (anaphase bridges, arrows) size bars: 20 µm.


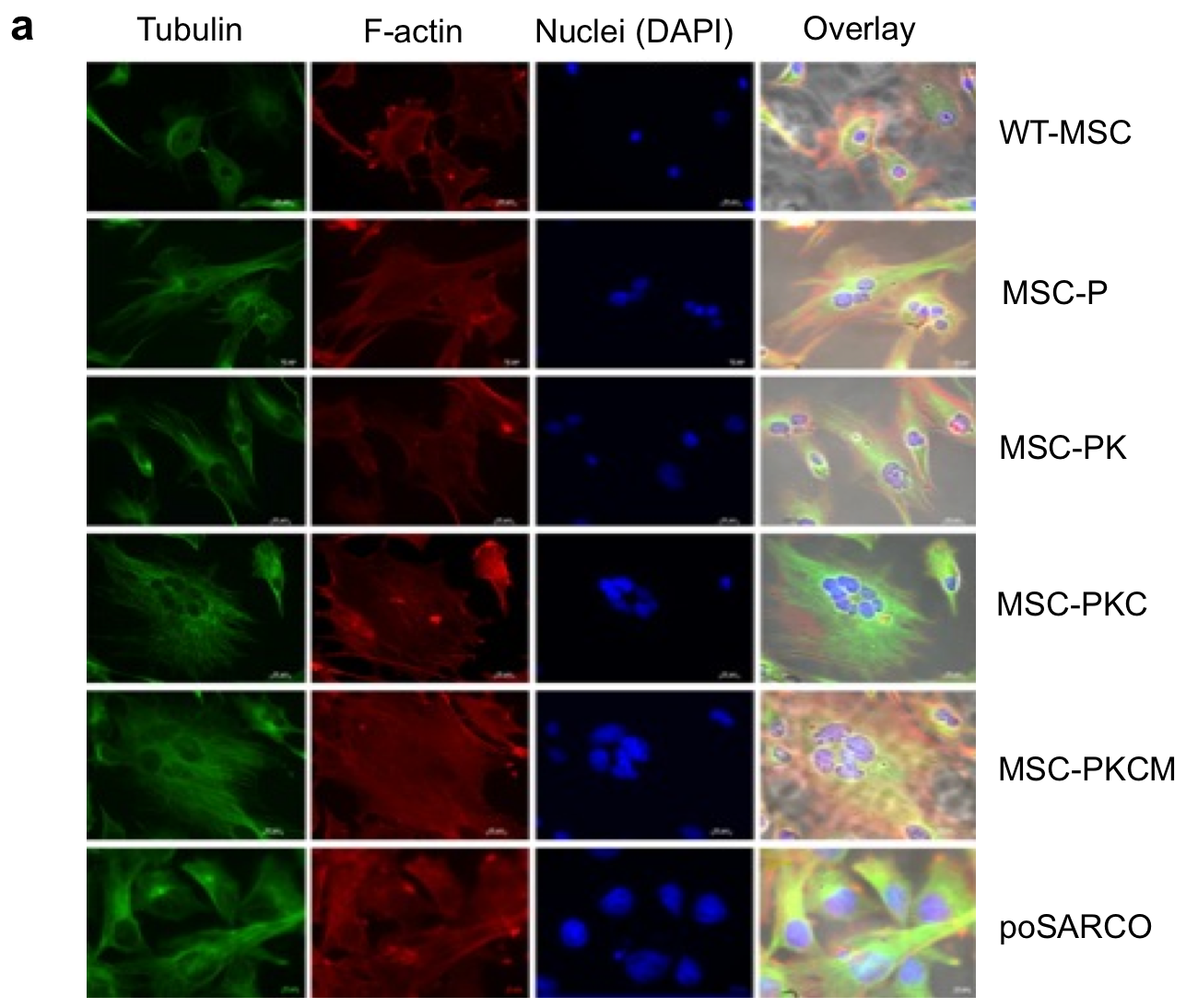


**
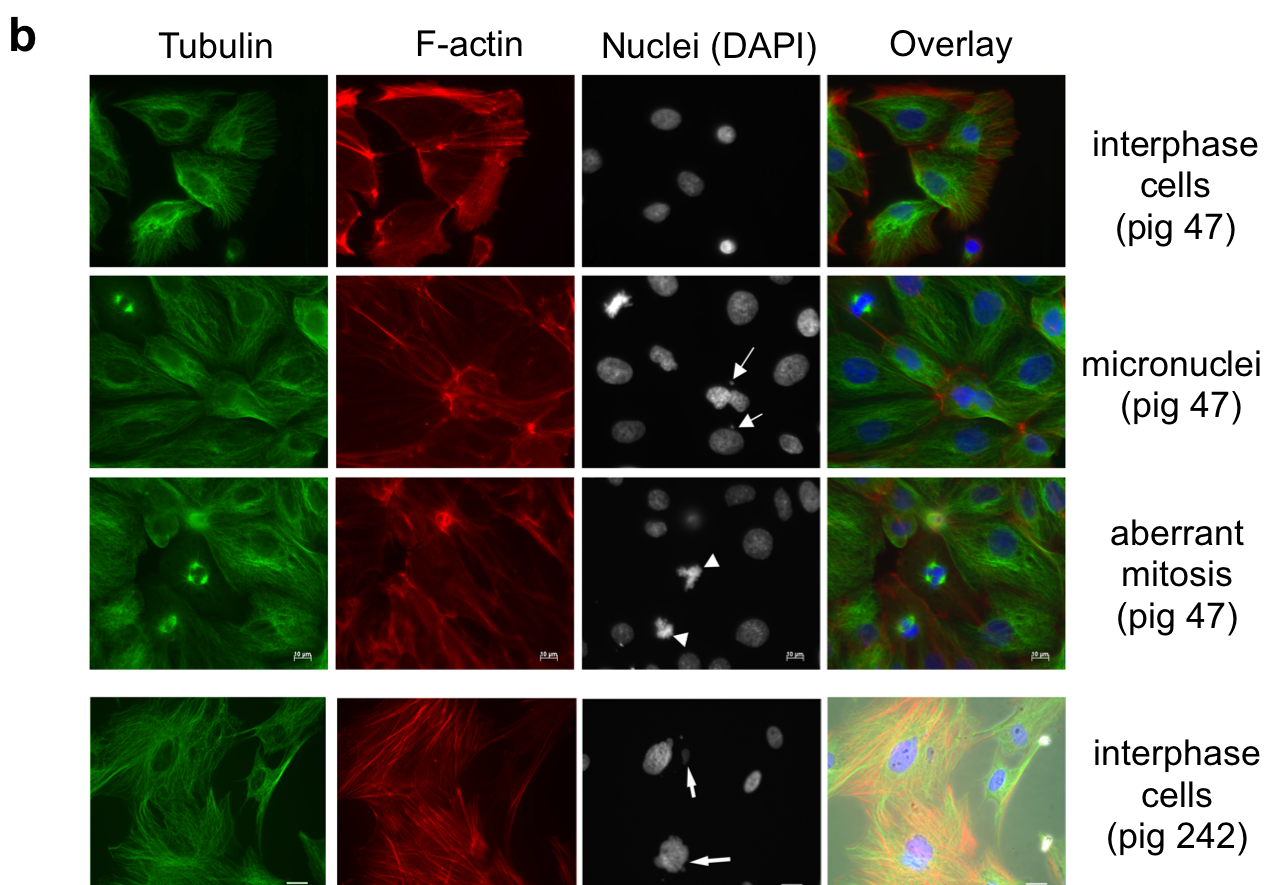
**

**
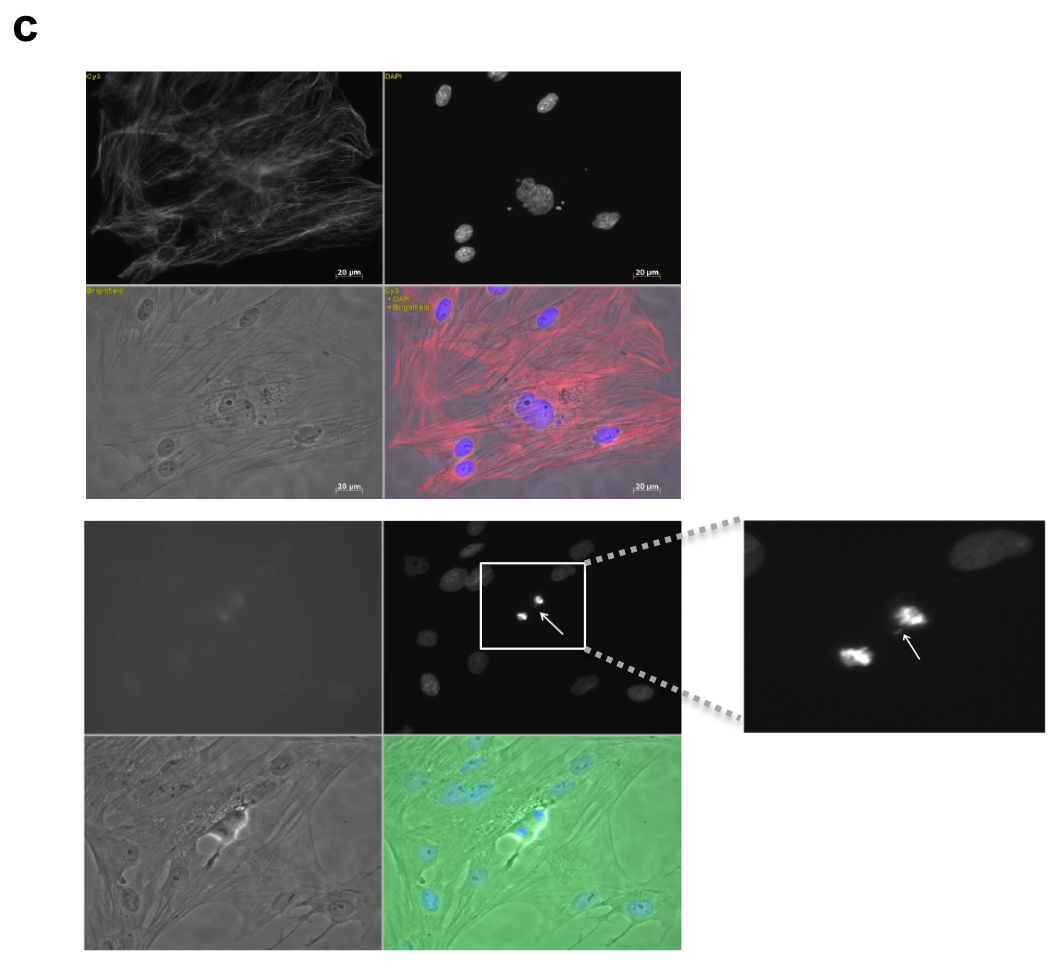
**

**Supplementary Tables**

**Supplementary Table 1. Gene expression profiling**

|  | **MSC-PK** | | **MSC-PKC** | | **MSC-PKCM** | | **poSARCO** | |
| --- | --- | --- | --- | --- | --- | --- | --- | --- |
| **Cell transformation** | | | | | | | | |
| Gene | p-value | Fold-change | p-value | Fold-change | p-value | Fold-change | p-value | Fold-change |
| AURKA | 1.97E-15 | 1.05E+01 | 8.14E-14 | 7.94E+00 | 3.82E-15 | 9.53E+00 | 2.05E-15 | 7.62E+00 |
| AURKB | 5.51E-13 | 2.11E+01 | 5.99E-12 | 1.85E+01 | 6.12E-13 | 2.06E+01 | 3.14E-13 | 1.54E+01 |
| BUB1 | 7.92E-13 | 4.13E+01 | 1.92E-11 | 2.93E+01 | 2.25E-12 | 3.23E+01 | 5.80E-13 | 2.68E+01 |
| BUB1B | 1.01E-12 | 5.87E+01 | 3.67E-11 | 3.65E+01 | 4.42E-12 | 4.04E+01 | 8.59E-13 | 3.53E+01 |
| BUB3 | 6.34E-05 | 1.70E+00 | 2.32E-02 | 1.32E+00 | 9.68E-05 | 1.66E+00 | 4.78E-05 | 1.60E+00 |
| MAD1L1 | 3.31E-03 | -1.97E+00 | 6.38E-04 | -2.53E+00 | 5.62E-03 | -1.87E+00 | 3.46E-04 | -2.16E+00 |
| MAD2L1 | 1.05E-08 | 1.39E+01 | 6.62E-07 | 8.72E+00 | 4.29E-08 | 1.08E+01 | 3.64E-08 | 8.03E+00 |
| MAD2L2 | 2.80E-07 | 1.62E+00 | 1.17E-06 | 1.62E+00 | 5.81E-05 | 1.36E+00 | 2.96E-05 | 1.33E+00 |
| CDC20 | 9.23E-10 | 1.78E+01 | 5.47E-09 | 1.72E+01 | 1.30E-09 | 1.66E+01 | 6.39E-10 | 1.29E+01 |
| ESPL1 | 2.07E-14 | 1.87E+01 | 3.28E-14 | 2.40E+01 | 3.42E-15 | 2.67E+01 | 2.51E-15 | 1.82E+01 |
| PTTG1 | 1.95E-09 | 1.66E+01 | 1.02E-07 | 1.07E+01 | 1.15E-08 | 1.20E+01 | 2.42E-09 | 1.10E+01 |
| **Telomere maintenance** | | | | | | | | |
| TERT | 8.13E-01 | 1.03E+00 | 5.36E-01 | -1.10E+00 | 2.13E-02 | -1.40E+00 | 5.30E-02 | -1.27E+00 |
| FEN1 | 2.59E-10 | 1.43E+01 | 1.67E-09 | 1.38E+01 | 7.34E-11 | 1.81E+01 | 9.20E-11 | 1.18E+01 |
| FANCD2 | 6.53E-10 | 1.20E+01 | 1.57E-08 | 9.30E+00 | 3.91E-09 | 9.02E+00 | 3.90E-10 | 9.31E+00 |
| FANCA | 1.79E-03 | 1.94E+00 | 1.17E-04 | 2.73E+00 | 4.00E-05 | 2.71E+00 | 4.36E-06 | 2.84E+00 |
| RAD51 | 2.18E-12 | 5.87E+00 | 1.78E-10 | 4.39E+00 | 3.21E-12 | 5.61E+00 | 1.69E-12 | 4.75E+00 |
| RAD51D | 2.56E-03 | 1.27E+00 | 3.34E-04 | 1.41E+00 | 8.98E-07 | 1.68E+00 | 3.24E-06 | 1.50E+00 |
| RAD52 | 5.34E-01 | -1.06E+00 | 2.19E-01 | 1.15E+00 | 7.34E-01 | -1.03E+00 | 1.60E-03 | 1.38E+00 |
| RAD54B | 6.34E-08 | 7.59E+00 | 4.89E-06 | 5.05E+00 | 3.88E-08 | 8.18E+00 | 4.55E-08 | 6.04E+00 |
| BRCA2 | 2.39E-07 | 3.66E+00 | 8.44E-05 | 2.43E+00 | 1.71E-07 | 3.78E+00 | 2.29E-08 | 3.80E+00 |
| MRE11A | 7.71E-06 | 2.16E+00 | 1.02E-03 | 1.70E+00 | 1.88E-05 | 2.04E+00 | 2.76E-06 | 2.06E+00 |
| BLM | 6.55E-14 | 2.19E+01 | 7.81E-12 | 1.26E+01 | 3.27E-14 | 2.52E+01 | 1.36E-14 | 1.92E+01 |
| WRN | 3.02E-04 | 3.31E+00 | 5.53E-02 | 1.83E+00 | 3.27E-04 | 3.27E+00 | 1.40E-04 | 3.07E+00 |
| POT1 | 5.93E-03 | 2.19E+00 | 3.92E-01 | 1.28E+00 | 2.00E-02 | 1.89E+00 | 7.99E-03 | 1.91E+00 |
| TERF1 | 1.59E-03 | 2.43E+00 | 9.82E-01 | 1.01E+00 | 1.44E-01 | 1.43E+00 | 4.51E-02 | 1.55E+00 |
| TERF2 | 3.34E-05 | 1.18E+00 | 2.57E-02 | 1.08E+00 | 6.12E-06 | 1.21E+00 | 5.09E-07 | 1.22E+00 |
| **Cell cycle control** | | | | | | | | |
| RB1 | 4.63E-01 | 1.24E+00 | 9.82E-02 | -1.75E+00 | 8.88E-01 | 1.04E+00 | 8.55E-01 | -1.05E+00 |
| E2F3 | 1.03E-06 | 2.56E+00 | 3.41E-01 | 1.14E+00 | 1.67E-07 | 2.94E+00 | 7.69E-06 | 1.99E+00 |
| CDK1 | 2.15E-08 | 1.73E+01 | 9.00E-07 | 1.12E+01 | 2.55E-08 | 1.67E+01 | 7.90E-09 | 1.42E+01 |
| CDK2 | 6.24E-12 | 5.88E+00 | 7.09E-10 | 4.24E+00 | 6.54E-12 | 5.85E+00 | 2.25E-11 | 4.09E+00 |
| CDK4 | 6.16E-08 | 1.54E+00 | 3.04E-06 | 1.43E+00 | 2.00E-07 | 1.48E+00 | 1.00E-07 | 1.43E+00 |
| CDK6 | 1.49E-08 | 2.06E+00 | 8.09E-06 | 1.64E+00 | 2.83E-07 | 1.79E+00 | 2.04E-09 | 2.05E+00 |
| CCNA1 | 7.18E-01 | -1.05E+00 | 3.11E-02 | 1.43E+00 | 6.41E-01 | -1.07E+00 | 5.04E-01 | 1.08E+00 |
| CCNA2 | 2.52E-11 | 3.87E+01 | 6.82E-10 | 2.65E+01 | 4.15E-11 | 3.43E+01 | 1.81E-10 | 1.61E+01 |
| CCNB1 | 8.13E-10 | 5.27E+01 | 1.26E-08 | 3.90E+01 | 1.27E-09 | 4.68E+01 | 2.08E-10 | 4.32E+01 |
| CCNB2 | 7.30E-14 | 7.25E+01 | 1.87E-12 | 4.86E+01 | 2.64E-13 | 5.16E+01 | 8.03E-14 | 3.99E+01 |
| CCNB3 | 7.34E-16 | 4.13E+01 | 2.41E-14 | 2.80E+01 | 1.62E-15 | 3.44E+01 | 4.31E-16 | 2.80E+01 |
| CCND1 | 4.00E-06 | 1.98E+00 | 3.98E-05 | 1.87E+00 | 9.57E-05 | 1.67E+00 | 1.65E-04 | 1.52E+00 |
| CCND2 | 1.41E-02 | 1.26E+00 | 1.58E-01 | 1.15E+00 | 8.58E-05 | 1.54E+00 | 9.63E-02 | 1.14E+00 |
| CCND3 | 3.83E-01 | -1.22E+00 | 3.84E-01 | -1.25E+00 | 6.63E-01 | 1.10E+00 | 4.20E-01 | 1.17E+00 |
| CCNE1 | 8.63E-08 | 2.20E+00 | 3.23E-04 | 1.55E+00 | 9.36E-08 | 2.19E+00 | 3.08E-05 | 1.53E+00 |

**Supplementary Table 1 (cont)**

|  | **MSC-PK** | | **MSC-PKC** | | **MSC-PKCM** | | **poSARCO** | |
| --- | --- | --- | --- | --- | --- | --- | --- | --- |
| **p53 target genes** | | | | | | | | |
| Gene | p-value | Fold-change | p-value | Fold-change | p-value | Fold-change | p-value | Fold-change |
| TP53 | 2.82E-08 | -6.33E+00 | 1.10E-03 | 2.27E+00 | 2.31E-03 | 1.95E+00 | 1.62E-05 | 2.64E+00 |
| MDM2 | 4.92E-06 | -3.10E+00 | 2.11E-07 | -5.05E+00 | 3.08E-05 | -2.62E+00 | 1.84E-06 | -2.89E+00 |
| TP63 | 1.64E-01 | 1.52E+00 | 9.27E-02 | 1.77E+00 | 7.19E-01 | 1.11E+00 | 1.57E-02 | 1.95E+00 |
| CDKN1A | 5.17E-13 | -5.98E+00 | 4.03E-12 | -5.75E+00 | 3.93E-13 | -6.17E+00 | 5.14E-13 | -4.71E+00 |
| CDKN1B | 4.59E-01 | -1.12E+00 | 1.78E-01 | -1.27E+00 | 1.82E-02 | -1.49E+00 | 5.99E-02 | -1.31E+00 |
| GADD  45A | 4.57E-06 | -1.60E+00 | 3.49E-06 | -1.72E+00 | 8.05E-07 | -1.72E+00 | 1.33E-02 | -1.18E+00 |
| CCNG1 | 3.13E-07 | -2.28E+00 | 1.37E-06 | -2.27E+00 | 1.70E-08 | -2.77E+00 | 6.25E-06 | -1.75E+00 |
| **Apoptosis** | | | | | | | | |
| TP53 | 2.82E-08 | -6.33E+00 | 1.10E-03 | 2.27E+00 | 2.31E-03 | 1.95E+00 | 1.62E-05 | 2.64E+00 |
| BCL2 | 1.19E-03 | 1.79E+00 | 1.15E-02 | 1.60E+00 | 1.05E-02 | 1.54E+00 | 5.13E-04 | 1.74E+00 |
| BAX | 1.73E-04 | -2.48E+00 | 1.74E-03 | -2.19E+00 | 3.08E-05 | -2.92E+00 | 3.72E-06 | -3.04E+00 |
| NOXA1 | 1.61E-01 | 1.25E+00 | 7.52E-02 | 1.38E+00 | 3.74E-01 | 1.15E+00 | 5.09E-01 | -1.09E+00 |
| BBC3 | 3.99E-07 | -4.95E+00 | 2.43E-06 | -4.72E+00 | 6.07E-06 | -3.62E+00 | 7.76E-08 | -4.79E+00 |
| BID | 2.52E-06 | 1.74E+00 | 2.84E-03 | 1.36E+00 | 1.51E-07 | 1.99E+00 | 1.95E-03 | 1.28E+00 |
| CASP9 | 3.92E-01 | -1.19E+00 | 7.47E-01 | 1.08E+00 | 1.32E-01 | 1.37E+00 | 1.25E-01 | 1.32E+00 |
| CASP3 | 7.41E-03 | 1.67E+00 | 2.69E-01 | 1.24E+00 | 3.12E-02 | 1.49E+00 | 3.52E-01 | 1.15E+00 |
| CASP6 | 6.71E-01 | -1.07E+00 | 4.19E-02 | -1.51E+00 | 9.80E-01 | -1.00E+00 | 1.39E-01 | -1.25E+00 |
| CASP7 | 1.37E-01 | 1.38E+00 | 5.60E-01 | 1.15E+00 | 4.51E-01 | 1.17E+00 | 2.25E-01 | -1.25E+00 |
| FAS | 6.37E-07 | -1.06E+01 | 4.35E-07 | -1.52E+01 | 1.41E-07 | -1.41E+01 | 1.08E-06 | -7.13E+00 |
| FASLG | 5.96E-01 | -1.07E+00 | 1.12E-01 | 1.27E+00 | 8.91E-01 | 1.02E+00 | 6.20E-01 | 1.06E+00 |
| TNFRS  F10B | 1.66E-02 | -1.19E+00 | 7.27E-05 | -1.47E+00 | 9.55E-01 | -1.00E+00 | 8.44E-01 | -1.01E+00 |
| TNFSF10 | 5.44E-03 | -1.13E+01 | 6.71E-03 | -1.38E+01 | 4.81E-03 | -1.18E+01 | 9.32E-03 | -6.91E+00 |
| FADD | 2.23E-03 | 1.40E+00 | 2.06E-04 | 1.65E+00 | 1.18E-05 | 1.79E+00 | 1.80E-05 | 1.63E+00 |
| CASP3 | 7.41E-03 | 1.67E+00 | 2.69E-01 | 1.24E+00 | 3.12E-02 | 1.49E+00 | 3.52E-01 | 1.15E+00 |
| CASP8 | 1.87E-04 | 2.32E+00 | 3.27E-02 | 1.58E+00 | 2.33E-03 | 1.88E+00 | 1.02E-02 | 1.55E+00 |
| **Chromosomal instability** | | | | | | | | |
| AURKA | 1.97E-15 | 1.05E+01 | 8.14E-14 | 7.94E+00 | 3.82E-15 | 9.53E+00 | 2.05E-15 | 7.62E+00 |
| AURKB | 5.51E-13 | 2.11E+01 | 5.99E-12 | 1.85E+01 | 6.12E-13 | 2.06E+01 | 3.14E-13 | 1.54E+01 |
| BUB1 | 7.92E-13 | 4.13E+01 | 1.92E-11 | 2.93E+01 | 2.25E-12 | 3.23E+01 | 5.80E-13 | 2.68E+01 |
| BUB1B | 1.01E-12 | 5.87E+01 | 3.67E-11 | 3.65E+01 | 4.42E-12 | 4.04E+01 | 8.59E-13 | 3.53E+01 |
| BUB3 | 6.34E-05 | 1.70E+00 | 2.32E-02 | 1.32E+00 | 9.68E-05 | 1.66E+00 | 4.78E-05 | 1.60E+00 |
| MAD1L1 | 3.31E-03 | -1.97E+00 | 6.38E-04 | -2.53E+00 | 5.62E-03 | -1.87E+00 | 3.46E-04 | -2.16E+00 |
| MAD2L1 | 1.05E-08 | 1.39E+01 | 6.62E-07 | 8.72E+00 | 4.29E-08 | 1.08E+01 | 3.64E-08 | 8.03E+00 |
| MAD2L2 | 2.80E-07 | 1.62E+00 | 1.17E-06 | 1.62E+00 | 5.81E-05 | 1.36E+00 | 2.96E-05 | 1.33E+00 |
| CDC20 | 9.23E-10 | 1.78E+01 | 5.47E-09 | 1.72E+01 | 1.30E-09 | 1.66E+01 | 6.39E-10 | 1.29E+01 |
| ESPL1 | 2.07E-14 | 1.87E+01 | 3.28E-14 | 2.40E+01 | 3.42E-15 | 2.67E+01 | 2.51E-15 | 1.82E+01 |
| PTTG1 | 1.95E-09 | 1.66E+01 | 1.02E-07 | 1.07E+01 | 1.15E-08 | 1.20E+01 | 2.42E-09 | 1.10E+01 |

**Supplementary Table 2. PCR primers used and diagnostic fragments amplified**

| **Primer name** | **Sequence** | **Product size** |
| --- | --- | --- |
| KRAStarg­F | 5’ ACGCGGGGAATGAGGAAT 3’ | 3.4 kb |
| KRAStargR | 5’ GAAAGACCGCGAAGAGTTTG 3’ |
| KRAStarg­F | 5’ ACGCGGGGAATGAGGAAT 3’ | 3.3 kb |
| KRASInt1R | 5’ TGAGGAAAAGAACAGTGCAAA 3’ |
| KRAStargLAF | 5’ CCAGCCATCTGTTGTTTGCC 3’ | 10.8 kb |
| KRAStargLAR | 5’ GAAGAAGGGACTGGGGTGTG 3’ |
| MutF | 5’ GAGCAACGGCTACAATCA 3’ | 2.8 kb |
| MutR | 5’ TGAAAAAGGACTGCACAGGA 3’ |
| KRASEx1F | 5’ CATTTCGGACTGGGAGCTA 3’ | 536 bp |
| bsR | 5’ GGCAGCAATTCACGAATC 3’ |
| KRASInt1F | 5’ AAAGCGGTACTTGCCTTTAAT 3’ | LSL: 1.5 kp  loxP: 201 bp  WT: 167 bp |
| KRASInt1R | 5’ TGAGGAAAAGAACAGTGCAAA 3’ |
| TP53Int1F | 5’ TGAGGAATTTGTATGCCAAGG 3’ | LSL: 1.9 kb  loxP: 254 bp  WT: 198 bp |
| TP53Int1R | 5’ TTCCACCAGTGAATCCACAA 3’ |
| KRASEx1F | 5’ CATTTCGGACTGGGAGCTA 3’ | 492 bp |
| KRASEx4R | 5’ TCCTGAGCCTGTTTTGTGTC 3’ |
| TP53Ex1F | 5’ GCAGGTAGCTGCTGGTCTC 3’ | 1.3 kb |
| TP53Ex11R | 5' AGGGACTTCAAAAGGGGATG 3' |
| BS-p16F | 5’ GGGGAGTAGTATGGAATTTT 3’ | 266 bp |
| BS-p16R | 5’ CAAAAAAAAAACTCCAACTC 3’ |
| CNV-KRASF | 5’ AGAGGGCTTGATAGCGTTTG 3’ | 251 bp |
| CNV-KRASR | 5’ GCCTGCACAAGTCAATATGC 3’ |
| CNV-GAPDHF | 5’ TAGGTTTGGGTTGGAACAGC 3’ | 233 bp |
| CNV-GAPDHR | 5’ AACCCAGTCTTGGTCAGTGG 3’ |
| RT-p16F | 5’ AACGCACCGAACCGTTAC 3’ | 176 bp |
| RT-p16R | 5’ AGGACCACCAAAGTGTCC 3’ |
| RT-p14F | 5’ CGTGCTGTTGCTAGTGAGGA 3’ | 234 bp |
| RT-p14R | 5’ AGGCGTCTCGCACGTCTA 3’ |
| RT-MycF | 5’ CCTCGGACTCTGCTCTCCT 3’ | 369 bp |
| RT-MycR | 5’ ATTTTCGGTTGTTGCTGATCTGT 3 ’ |
| RT-GAPDHF | 5’ TTCCACGGCACAGTCAAGGC 3’ | 576 bp |
| RT-GAPDHR | 5’ GCAGGTCAGATCCACAACC 3’ |
| RT-TERTF | 5’ TGAACTTCCCTGTGGAGGAC 3’ | 387 bp |
| RT-TERTR | 5’ GGAGGAAAAATGAGGGGTTC 3’ |
